# Heterosis Is Prevalent Among Domesticated but not Wild Strains of *Saccharomyces cerevisiae*

**DOI:** 10.1534/g3.113.009381

**Published:** 2013-12-16

**Authors:** Marcin Plech, J. Arjan G. M. de Visser, Ryszard Korona

**Affiliations:** * Institute of Environmental Sciences, Jagiellonian University, 30-387 Krakow, Poland; † Laboratory of Genetics, Wageningen University, Wageningen, the Netherlands

**Keywords:** genetic load, heterosis, sequence divergence

## Abstract

Crosses between inbred but unrelated individuals often result in an increased fitness of the progeny. This phenomenon is known as heterosis and has been reported for wild and domesticated populations of plants and animals. Analysis of heterosis is often hindered by the fact that the genetic relatedness between analyzed organisms is only approximately known. We studied a collection of *Saccharomyces cerevisiae* isolates from wild and human-created habitats whose genomes were sequenced and thus their relatedness was fully known. We reasoned that if these strains accumulated different deleterious mutations at an approximately constant rate, then heterosis should be most visible in F1 heterozygotes from the least related parents. We found that heterosis was substantial and positively correlated with sequence divergence, but only in domesticated strains. More than 80% of the heterozygous hybrids were more fit than expected from the mean of their homozygous parents, and approximately three-quarters of those exceeded even the fittest parent. Our results support the notion that domestication brings about relaxation of selection and accumulation of deleterious mutations. However, other factors may have contributed as well. In particular, the observed build-up of genetic load might be facilitated by a decrease, and not increase, in the rate of inbreeding.

Inbreeding depression, the loss of fitness of inbred individuals when compared to outbred ones, is observed across different taxa (Charlesworth and Charlesworth 1987). A cross between two inbred but unrelated organisms often helps to improve fitness of progeny. This phenomenon is known as heterosis and is ascribed either to creation of superior (overdominant) heterozygous loci or to reciprocal complementation of harmful mutations. Genuine and informative examples of overdominance have been found (Krieger *et al.* 2010; Johnston *et al.* 2013), but their frequency appears rather low given how ubiquitous heterosis is (Hedrick 2012). Much, and probably most, of heterosis is caused by complementation of deleterious mutations present in one parent by functional alleles contributed by the other (Charlesworth and Willis 2009). Therefore, the extent of heterosis will ultimately depend on the genetic load of populations, that is, their total decline in fitness if compared to an ideal population free of harmful mutations (Haldane 1937). Accumulation of the load is governed by several factors, including the rate and fitness effects of mutations, their dominance status, the size and structure of the population, and the prevalent mode of reproduction (Wang *et al.* 1999; Whitlock 2002; Glemin *et al.* 2003). Although it is straightforward to postulate that mutations will accumulate most easily when their effects are small and recessive and when populations are small or highly structured, the role of the mode of reproduction is more subtle. In sexual species, increasing the rate of outbreeding can actually inflate the load of mutations because they become less frequently exposed to selection in homozygotes (Bataillon and Kirkpatrick 2000; Whitlock *et al.* 2000). Similarly, switching to an asexual mode of reproduction can lead to hiding mutations in heterozygous loci and a significant enlargement of the genetic load (Haag and Roze 2007). It should also be mentioned that the location of deleterious mutations is not the only possible difference between unrelated organisms. A cross between such organisms may reveal any existing genetic incompatibilities and result in low fitness of hybrids, that is, outbreeding depression (Lynch 1991).

The organism studied here, *Saccharomyces cerevisiae* or the budding yeast, is a unicellular and typically diploid organism. Under so-called vegetative growth, it reproduces asexually through mitotic cell division. Deprived of nutrients, it undergoes meiosis and produces haploid spores that normally mate soon after germination and re-establish a diploid cell. For its closest relative, *Saccharomyces paradoxus*, it has been estimated that a meiotic division occurs approximately every 1000 mitotic divisions, and when this happens selfing is approximately 100-times more frequent than outcrossing (Tsai *et al.* 2008). Outcrossing is also rare in *S. cerevisiae*. An analysis of the *S. cerevisiae* genome sequence suggests that after the split with *S. paradoxus*, outcrossing occurred only once every 50,000 generations (Ruderfer *et al.* 2006). The effective size of the entire population *S. cerevisiae* is likely large, assuming that it is comparable to that of *S. paradoxus*, which was estimated at 8.6 × 10^6^ (Tsai *et al.* 2008). Thus, it appears that the genetic load of the budding yeast was, for a long time, controlled by selection acting on an effectively large population of diploid strains reproducing primarily through mitotic divisions with occasional sexual cycles involving self-fertilization. It used to be speculated that *S. cerevisiae* isolates found in nature could be just refugees from human-associated cultures (Mortimer 2000). However, it has been recently established that *S. cerevisiae* is a true “wild” species and that its domesticated lineages derive from the wild ones (Fay and Benavides 2005; Legras *et al.* 2007; Libkind *et al.* 2011; Wang *et al.* 2012). There are several reasons to believe that domestication was associated with the relaxation of selection and accumulation of deleterious mutations. First, the domesticated lines were likely to go through large reductions of population size and adaptation to new environments (Liti *et al.* 2009; Schacherer *et al.* 2009; Hyma *et al.* 2011). Small population size makes selection against deleterious mutations less effective, but so does linkage between deleterious and adaptive alleles (Hill and Robertson 1966). Furthermore, natural niches are probably more diverse and thus test more genes than those created by humans. In a new environment, the unused genes become vulnerable to mutational erosion (Kawecki *et al.* 1997). It is also possible that even those genes that remain required are generally less intensely purged of mutations if one accepts that selection weakens when habitat becomes less variable and especially less stressful (Hoffmann and Parsons 1991). Finally, the regime of frequent selfing was likely violated or even abandoned, thus preventing the exposure of deleterious mutations in the haploid phase. Many laboratory and industrial strains are kept under conditions that are prohibitive for sporulation. These strains are often difficult or impossible to sporulate, which suggests that the ability to undergo sexual cycle degenerates in human-made environments (Johnston 1994). An example of a likely joint action of these factors—smaller population size, benign and specific environment, possible adaptation, and asexual reproduction—has been provided by a study in which a laboratory strain of confirmed history of “domestication” was found to undergo an accelerated rate of molecular evolution, demonstrating considerable relaxation of the purifying selection (Gu *et al.* 2005).

In this study, we used a collection of natural isolates of *S. cerevisiae* that were formerly subject to whole-genome analysis, and thus their phylogenetic distances (measured by the number of nucleotide substitutions) are known (Liti *et al.* 2009). Genetic diversity and phenotypic diversity present in these strains are remarkably high, encouraging their further use in genetic and ecological studies. A subset of these strains was utilized in an extensive mapping of quantitative trait loci for fitness-related traits in different environmental conditions (Cubillos *et al.* 2011; Warringer *et al.* 2011). Another subset was used in a study of dominance and found that heterosis was present but limited in this sense that average progeny fitness almost never exceeded that of the best parent (Zorgo *et al.* 2012). We sought to expand the latter analysis. Our sample involved approximately twice as many parental strains from the collection of Liti *et al.* (2009) and about six times more unique hybrid strains. Crucially, the large sample allowed us to compare two major groups, those found in environments associated with humans (collectively called “domesticated”) and the rest which were isolated from natural habitats (“wild”). We found that heterosis was present among domesticated, but not among wild, strains. Heterosis among domesticated strains was positively correlated with the phylogenetic distance between parental strains, suggesting that multiple mutations accumulated during domestication were responsible.

## Materials and Methods

### Strains

We used a formerly described collection of strains that had been subject to whole-genome sequencing (Liti *et al.* 2009). Each originally diploid strain was sporulated and the resulting tetrads of haploid spores were dissected (Cubillos *et al.* 2009). Because the spore cell contained an active allele of the *HO* gene, cells descending from it switched the mating types, mated, and formed homozygous diploids, except for the *MAT* locus. Subsequent replacement of *HO* by the *hph*MX4 cassette (coding for resistance to hygromycin B), followed by sporulation and tetrad dissection, produced isogenic haploids of stable **a** or *α* mating type. Another gene in these strains, *URA3*, had been substituted with the *kan*MX4 cassette coding for selectable marker resistance to kanamycin (Cubillos *et al.* 2009). We replaced the *hph*MX4 with *nat*MX4 cassette in the *α* strains. We then mated the **a** and *α* haploids in all possible combinations either in isogenic or nonisogenic pairs. An important consequence of the described procedure is that the strains we prepared to work with were not genetically intact isolates. The original wild strains were diploids, but some of their loci were possibly heterozygous. We used haploid genotypes derived from particular wild isolates to make either homozygous or heterozygous diploids (the marker loci were *ho*::*hph*MX4/*ho*::*nat*MX4 and u*ra3*::*kan*MX4/*ura3*::*kan*MX4 in all strains).

**Figure 1:**
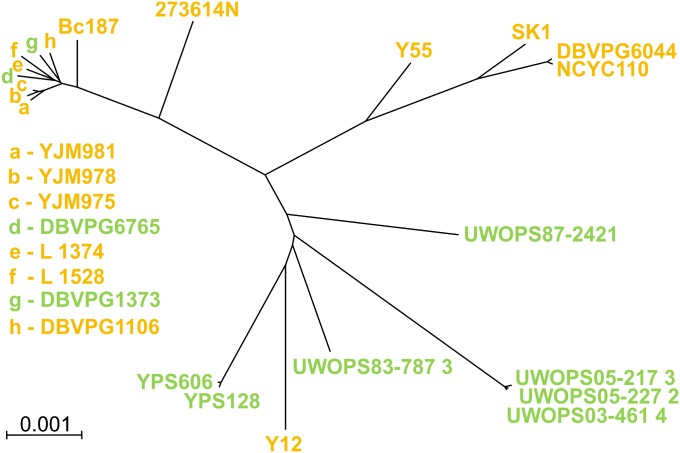
Phylogenetic relations between strains used in this study. Colors indicate domesticated (yellow) and wild (green) yeasts. Phylogenetic distances are based on differences in the number of nucleotide substitutions as in the study by Liti *et al.* (2009).

We originally intended to use all available strains but could not use a few because of difficulties in mating or strong aggregation in liquid cultures, which made estimation of growth rate unreliable. We worked with 22 strains, 9 of which were “wild,” originating from natural habitats, and the remaining 13 were “domesticated,” coming from laboratories, agriculture, industry, or human patients (Figure 1). The distinction between domesticated and wild strains was made by the authors of the original study (Liti *et al.* 2009). After completing all possible matings, we obtained 22 homozygous diploid strains and 231 heterozygous diploid strains representing all possible combinations among the 22 strains.

### Liquid cultures

Fitness was estimated in two ways. First, we measured the maximum growth rate in liquid culture. We used the nutritionally rich YPD medium with glucose in most fitness assays. It served as a base environment in which single chemicals were added or physical factors were modified to create test environments. In addition to the broth-based media, we used typical minimal medium (synthetic dextrose). While selecting test environments, we attempted to create conditions known to induce a range of phenotypes known to differ in the expression of a large number of genes (Hampsey 1997).

Fresh medium was dispensed into aliquots of 200 μl contained in wells of a standard flat-bottom titration plate. Microcultures were initiated by inoculating each aliquot with 1 μl of an overnight culture of each strain. The plates were incubated with agitation of 1250 rpm and wide-band optical density (OD) readouts were measured every 20–60 min with the Infinite m200 Microplate Reader (Tecan). The OD readouts were corrected for background effects, log-transformed, and used to determine the range of exponential growth. This was performed by finding lower and upper limits of OD, between which the Pearson *r* was highest when averaged over all cultures in the same test environment. This usually yielded regressions based on no less than five time points and an *r* of 0.999, with rare cases of an *r* < 0.99. Maximum growth rate (MGR) was then estimated from the slope of the linear regression of Log OD *vs.* time. Every strain was assayed independently several times (heterozygotes were usually assayed four times; homozygotes were usually assayed six times). For nine of the 11 test environments, the temperature was set at 30°. These included synthetic dextrose (synthetic medium with 2% glucose supplemented with uracil), YPG (nutrient-rich medium with 3% glycerol as a source of carbon), YPGal (nutrient-rich with 2% galactose), and YPD (nutrient-rich with 2% glucose). The remaining five media were based on YPD with the following supplements added: benomyl (40 μg/ml); DMSO (6%); NaCl (2%); salicylate (2%); and ZnSO_4_ (0.5 mg/ml). There were also two environments with no supplements but altered temperatures, YPD 20° and YPD 35°.

### Agar cultures

Using increasing doses of NaCl, ZnSO_4_, and salicylate, we created gradients of stresses up to a point at which some strains stopped growing. We detected a number of strains that made no more than approximately three cell doublings, even though other patches continued to grow. We decided to classify patches showing, on average, no more than three cell doublings as nongrowing and set their fitness to zero. We reasoned that because the cells used for inoculation were raised under benign conditions, their initial growth possibly resulted from carry-over effects from these pregrowth conditions. We divided the strains into “growers” and “nongrowers;” growers were those that had at least one positive fitness score out of three trials.

We measured the ability to grow under extreme stress conditions on agar plates. In these harsh environments, measuring the maximum growth rate in liquid culture was not feasible. Assays were started by inoculating agar surfaces with 1.5 μl drops of stationary phase cultures that were highly synchronized by a strictly parallel regime of inoculation and overnight incubation in YPD. After drying out, the drops left round patches of yeast cells that were immediately photographed, transferred to 30°, and then photographed again at the end of incubation. The duration of incubation varied depending on particular stress conditions as follows: NaCl 2% (48 hr), 8% (72 hr), 9% (96 hr), and 10% (120 hr); Na-salicylate 2.5% (24 hr), 3.5% (24 hr), 4% (48 hr), and 5% (72 hr); ZnSO_4_ 0.5 mg/ml (24 hr), 2 mg/ml (48 hr), 2.5 mg/ml (72 hr), and 2.75 mg/ml (120 hr). The photographs were analyzed with the Colonyzer 2.0 computer program (Lawless *et al.* 2010), which returns estimates of “intensity,” which is a measure of light reflected by yeast patches. To calibrate the intensity scores, we carved out a random sample of 40 patches and counted the number of cells within each of them. The relation between the two scores turned out to be linear and significantly correlated (*r* = 0.986) over the entire range of patch sizes that were analyzed (Supporting Information, Figure S1). As a measure of fitness, we used the log ratio of final and initial cell numbers divided by the time of incubation. This parameter signified the mean growth rate per hour over the entire period of incubation and included an initial lag phase followed by a phase of growth in which the rate of cell doubling was decelerating.

## Results

### Determinants of variation in fitness

**Figure 2:**
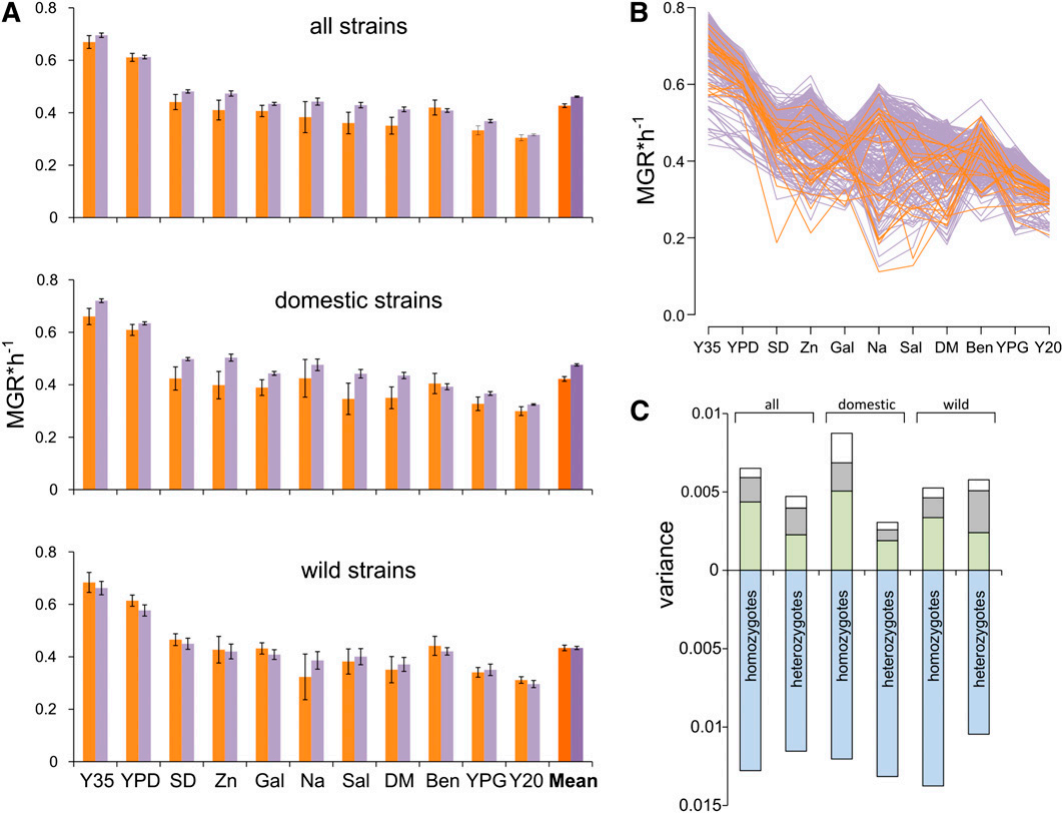
Determinants of phenotypic variation. Fitness was estimated as the maximum growth rate (MGR) in 11 test environments, here ordered according to descending average MGRs of heterozygote strains. (A) Mean MGRs in 11 environments calculated for all strains as well as for domesticated and wild strains separately. Colors are used to distinguish between homozygous (orange) and heterozygous (violet) strains. (B) MGRs of individual strains in particular environments. Colors are indicative as already noted. (C) Components of variance (absolute values of *σ^2^* estimates) attributable to the effect of environment (blue), genotype (gray), genotype–environment interaction (green), and error term (white). In this and other figures, SD is an abbreviation for minimal glucose medium. Other media include plain YPD at 30° (YPD) and its modifications as follows: 35° (Y35); ZnSO_4_ (Zn); galactose (Gal); NaCl (Na), salicylate (Sal); DMSO (DM); benomyl (Ben); glycerol (YPG); and 20° (Y20).

We used MGR measured in liquid culture as a proxy for fitness. The estimates are listed in Table S1. Individual strains were measured four times in every environment; the intraclass correlation coefficient (*ICC*) was 0.920 when averaged over all 11 environments. MGRs varied widely among environments. This was expected because they represented different suboptimal conditions. More relevant was the question whether MGR of heterozygotes was on average higher than that of homozygotes (it was, as shown in Figure 2A). The 231 hybrids were more fit than the 22 homozygotes from which they were derived (F = 95.35; df = 1 and 251; *P* < 0.0001; ANOVA, *aov* function in R-base). Considering only the domesticated strains, we again found that the 78 heterozygotes had a higher mean MGR than the 13 homozygotes (F = 125.9; df = 1 and 89; *P* < 0.0001), but there was no such difference when the 9 homozygotes and 36 heterozygotes derived from the wild isolates were compared (F = 1E−04; df = 1 and 43; *P* = 0.9914). These observations could suggest that the domesticated and wild strains were much different in their average MGR. However, this was not the case; respective means (homozygote fitness averaged across all media) were 0.4337 and 0.4222 for wild and domesticated strains, and the difference was not significant (t = 1.6165, df = 1279.34; *P* = 0.9469).

We then asked to what extent MGR was determined by the environment, genes, and interactions between these two factors. Figure 2B shows that MGR of individual strains fluctuated between the test environments. To quantify these effects, we calculated components of variance as defined in standard ANOVA models (R’s *lmer* function of *lme4* library). Figure 2C shows that, as expected, most variation can be attributed to differences between environments; in favorable environments all strains grew well and in unfavorable ones all performed poorly. More interestingly, genotype–environment interaction had a generally higher impact on fitness variation than the genotype of the strain. This result was important because it confirmed that the environments used in this study were indeed sufficiently diverse to uncover different components of the genetic variation. Another striking finding is that the impact of strains and that of genotype–environment interaction was considerably smaller among heterozygotes than among homozygotes for the domesticated strains; these strains were phenotypically more homogeneous after hybridization. A general conclusion emerging from these comparisons is that the average fitness of heterozygous strains was higher than that of homozygous strains only in domesticated, but not wild, strains.

### Mid-parent and best-parent heterosis

**Figure 3:**
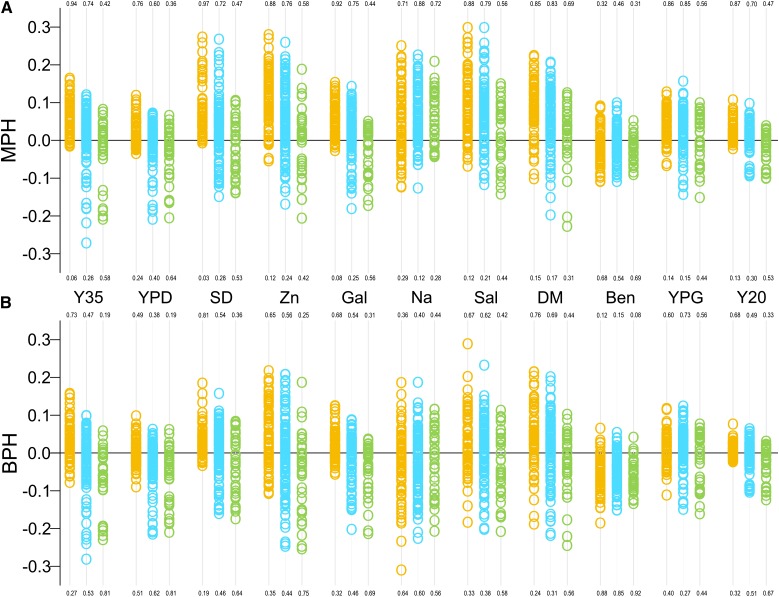
Mid-parent heterosis (A) and best-parent heterosis (B). Plots illustrate distributions and numbers show proportions of positive and negative scores of MPH or BPH. Colors refer to crosses between two domesticated (yellow), domesticated and wild (blue), and two wild strains (green).

For every heterozygous stain, we calculated the mid-parent heterosis (MPH) as the difference between its MGR and the average MGR of its parental homozygous strains. Best-parent heterosis (BPH) was calculated by subtracting MGR of a heterozygote from MGR of its most fit parental homozygote. Figure 3 shows that MPH was frequent within, but differed between, three groups of heterozygotes derived from crosses: between two domesticated strains, between domesticated and wild strain (“mixed”), and between two wild strains. There is a regular pattern with domesticated heterozygotes showing the largest heterosis, mixed hybrids showing intermediate heterosis, and wild hybrids showing no heterosis. Averaged over 11 environments, MPHs of the three groups were 0.055, 0.032, and −0.003, respectively. Differences between averages were significant globally (*F* = 92.653; *df* = 2 and 254; *P* < 0.001) and for all three in-pair comparisons (not shown). Heterosis was especially apparent for the domesticated heterozygotes; as many as 82% of them were better than predicted by their parental averages. The wild strains were approximately equally better than (51%) and worse than expected (49%). BPH was less frequent than MPH, but again was most pronounced among the domesticated strains. The scatterplot distributions presented in Figure 3 and the aforementioned statistical comparison point to the domesticated and wild heterozygotes as two groups that clearly differ in the extent of heterosis. Several of our subsequent analyses will focus on this intriguing difference.

In a previous study of heterosis, six domesticated strains and three wild strains were used (Zorgo *et al.* 2012). The study reported frequencies of MPH for each of the nine strains (pooled over all its crosses and all tests environments). The scores for wild and domesticated strains were intermixed, and thus no significant difference between the two groups could be found. We could not find statistically significant differences in our data if only these nine strains were taken into account average MPHs for the wild and domestic strains over 11 test environments were 0.045 and 0.059 (*t* = 0.988; *df* = 20; *P* = 0.335). Thus, our expansion of the sample size was critical to find differences in heterosis between the wild and domesticated strains.

### Sequence divergence and heterosis

**Figure 4:**
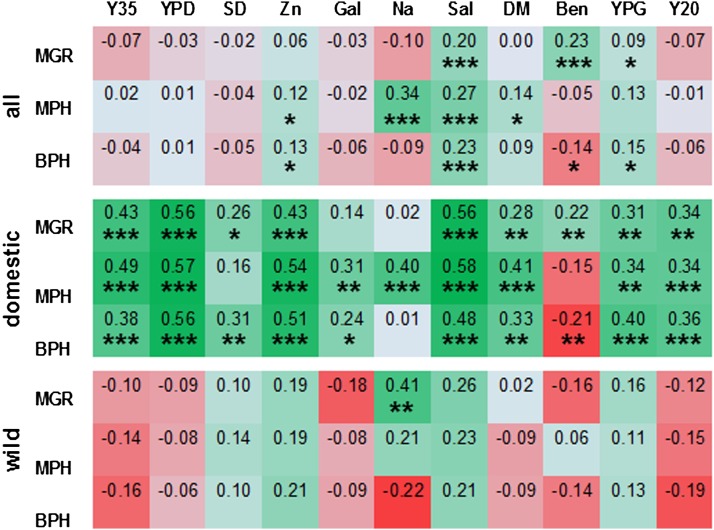
Correlations between hybrid fitness and the sequence divergence between parents. Fitness is expressed as absolute values of hybrid MGRs or as parameters related to parental maximum growth rate (MPH and BPH). Color intensities indicate strength of correlations, either positive (green) or negative (red). Statistical significance of the calculated Pearson *r* coefficients is indicated by asterisks as follows: *0.05; **0.01; and ***0.001.

Figure 1 shows the sequence divergence between our strains based on differences in the number of nucleotide substitutions (Liti *et al.* 2009). We reasoned that the more distant parents are, the higher the number of different accumulated mutations and therefore the more likely heterosis is by reciprocal compensation. Figures S2, Figure S3, and Figure S4 show how phylogenetic distance correlated with one of three measures of heterozygote fitness, absolute MGR and the two indexes of heterosis, MPH and BPH. This analysis is summarized in the main text in the form of Pearson correlation coefficients presented in Figure 4. Not surprisingly, the three measures of hybrid fitness often, although not always, followed similar patterns. Pearson correlations with the distance were weak and either positive or negative when all strains or only the wild strains were inspected. Crucially, correlations became much stronger and generally positive when only the domesticated strains were considered.

### Heterozygotes and homozygotes under extreme stress

**Figure 5:**
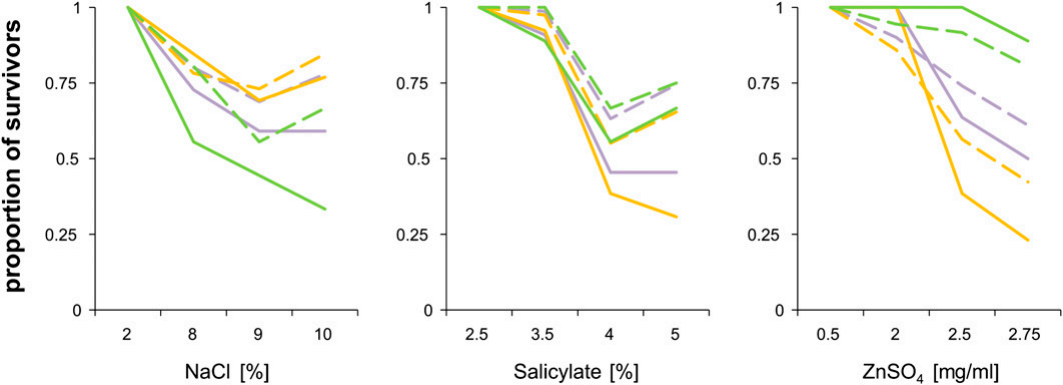
Viability under increasing stress. The proportion of survivors among homozygous (solid lines) and heterozygous strains (dashed lines) is shown. Colors refer to all (violet), domesticated (yellow), and wild (green) strains.

In the test environments described, growth was slowed when compared with YPD (Figure 1), but all homozygotes and heterozygotes were able to grow. We extended our tests of heterosis under more stressful conditions when growth ceases for some strains. Table S2 lists strains that were not able to grow and estimates of the growth rate for those that did grow. The rate of growth estimated on agar plates was not the same as MGR measured in liquid cultures (see *Materials and Methods*). Figure 5 shows that strains able to grow were generally more frequent among heterozygotes than among homozygotes. An opposite tendency was noted only for the wild strains in the ZnSO_4_ gradient, but in this case both homozygous and heterozygous strains were affected little and the difference in the fraction of growers was small.

We then asked whether fitness of a heterozygous strain depended on the phylogenetic distance between parental strains. To this end, growth rates attained under each of the three stresses were correlated with the sequence divergence between their parents. In contrast to the benign conditions (Figure 4), no statistically significant correlations with sequence divergence were observed for any stress, irrespective of whether they were calculated for domesticated or wild strains (not shown).

**Figure 6:**
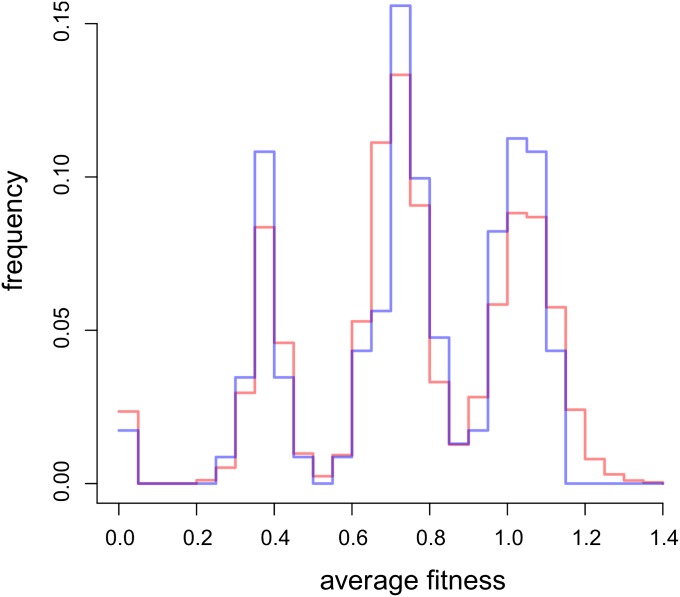
Distribution of fitness averaged over the three most extreme stresses. Fitness was normalized by dividing individual growth rates by the stress-specific median growth rate of viable strains. An empirical distribution (blue) is confronted with the expected one (red).

We hypothesized that some strains could be more resilient to any extreme environments and that others could be more affected by unusual environmental pressures. In such instances, we would observe overrepresentation of lines not growing or growing very well in all three stressful environments. However, the obtained distribution had four modal peaks, reflecting the fact that some strains did not grow in any of the tested environments whereas others grew well in one, two, or three of them. To obtain an expected distribution for growth rates that were uncorrelated across stress environments, we drew at random (10,000 times) a fitness estimate from each of the three environments and calculated the average. The empirical and expected distributions were strikingly alike (Figure 6) and there was no statistically significant difference between them (Kolgomorov-Smirnov test; D = 0.0756; *P* = 0.15). It thus appears that being (un)fit under some extreme conditions is not predictive for performance in other harsh environments.

We conclude that there were signs of heterosis under strong environmental stress, because heterozygotes were more likely to sustain it than were homozygotes. Unlike that observed in less stressful conditions, the fitness of heterozygotes did not depend on the genetic divergence between their parents. There was not any visible tendency of some strains to perform better than others in all three extreme environments (Figure 6), whereas there was a substantial “strain” component in the less stressful environments (Figure 2C). We explain these differences in the *Discussion*.

## Discussion

The beneficial effect of heterozygosity was evident only for strains of domestic origin. In these strains, heterosis was strong (with heterozygote fitness often higher than that of the fittest parent), evident in multiple environments, and positively correlated with the sequence divergence between parental strains. In striking contrast, no average advantage of heterozygotes over homozygotes was observed among the wild isolates, nor was there a significant relation between heterosis and sequence divergence in these strains. Thus, we conclude that domestication of the budding yeast was associated with accumulation of deleterious mutations similar to that in plants and animals (Eyre-Walker *et al.* 1998; Diamond 2002; Doebley *et al.* 2006; Lu *et al.* 2006; Cruz *et al.* 2008). A universal factor facilitating accumulation of slightly deleterious mutations is reduction of the effective population size. Although it appears reasonable to assume that the effective size of domestic populations decreased as they experienced passing through small numbers of cells in laboratory and industrial propagation or during infections, actual estimates are lacking. Neither the genetic variation within nor the migration rate between local population can be compared for domesticated and wild yeast. This is because, typically, a single strain from a place of isolation is further studied, carrying only a fraction of genetic variance present in the population of its origin. It is likely, however, that local yeast populations will soon be sampled for multiple clones and that whole-genome comparisons within and between populations will be used to derive required parameters. There is more evidence for another potential factor promoting accumulation of genetic load, namely, a decrease in the rate of selfing, which is typical for wild yeast and makes selection more effective by combining recessive deleterious mutations to homozygotes (Zeyl 2009; Masel and Lyttle 2011). Recent studies have found that domesticated strains do have higher proportions of heterozygous loci than the wild ones (Diezmann and Dietrich 2009; Magwene *et al.* 2011; Wang *et al.* 2012). Heterozygous loci can arise through mutations that appear and persist in lineages reproducing asexually (mitotically). Another study involving whole-genome sequencing has demonstrated, however, that domesticated strains may also undergo more frequent outcrossing and recombination (Magwene *et al.* 2011). Whether and how much selfing is replaced by outcrossing and whether and how much sexual reproduction is replaced by asexual reproduction in domesticated strains are likely to be tested more thoroughly as genomic studies proliferate.

Domesticated populations would be less intensely purged of mutations if selection coefficients were generally lower. This could occur if human-made habitats were generally more benign than the wild ones. There is abundant literature postulating that phenotypic effects of many mutations are dampened when the environment is benign and amplified when it is stressful (de Visser *et al.* 2003). If this is universally true, then the pattern observed by us under benign and lightly stressful environments should be repeated and possibly enlarged under extreme stress. However, whereas heterosis was seen under strong stress, it was not larger for domesticated strains and there was no correlation between heterosis and the phylogenetic distance between parents. One possible explanation is that in both human-made and wild habitats, semi-lethal conditions are rare and thus the effect of selection is scarce, or it could be that the harsh stresses actually experienced by yeast are different from those tested here. Alternatively, selection seems strong enough for neither domesticated nor wild strains to accumulate sizable amounts of stress-dependent mutations. More formally, selection coefficients of mutations exposed by stress are much larger than the reciprocal of the effective population sizes typical for most habitats, both wild and domesticated. If so, then the observed genetic variation would be caused by relatively few and recent mutations and thus the correlation between heterosis and phylogenetic distance would not develop. It is also possible that the mutational effects pertinent to moderately and extremely difficult conditions are substantially different. The observed decreases in the growth rate under benign conditions were likely caused by malfunctioning of specific enzymes. Damages hampering metabolic fluxes are effectively compensated in heterozygotes, guaranteeing an emergence of heterosis if the compensation is reciprocal (Kacser and Burns 1981). In contrast, when growth is barely possible, it is possible that an overall burden of damages matters, rather than single mutations, and the phenotypic reaction may be more threshold-like than incremental. Although these interpretations are hypothetical, our results are in accordance with the emerging consensus that the effect of stress does not lead to the simple amplification of phenotypes observed under benign conditions (McGuigan and Sgro 2009; Agrawal and Whitlock 2010). Moreover, our results are in accordance with those obtained in a former study in which 16 strains belonging to the same collection as our strains were used in media supplemented with high doses of alcohol or maintained at extreme temperatures. Hybrids were only moderately, and not universally, better than parental strains (Timberlake *et al.* 2011).

Our claim that most of the observed heterosis results from complementation of deleterious mutations would be most convincingly supported by pointing to actual deleterious substitutions or, at least, by estimating how numerous such substitutions can be. The number of nonsynonymous substitutions can be very large, and it was originally estimated at 24,418 for the whole collection (Liti *et al.* 2009). Substitutions that contributed to the phenotypic variation observed in our assays were possibly much fewer. Some of the strains used here have been recently reanalyzed with an accuracy that allowed listing of individual substitutions reliably. Mutations causing a likely loss of function (LOF), such as frame-shifts and premature stop codons, were scored and the data were kindly provided to us (A. Bergström and G. Liti, personal communication). In four wild stains used by us, there were 89.3 ± 21.3 LOF mutations (mean ± SD). A very close score, 93.5 ± 4.4, was obtained for 11 of the domesticated strains we used. Differences were not larger if we restricted our comparisons to verified ORFs or essential genes (the latter were very sparse). These results suggest that the observed heterosis cannot be explained by complementation of LOF mutations. However, this claim is not strong because it could be that only some of the detected LOF mutations matter while most of the others have little effect. Even essential genes can differ; in two of the compared strains of *S. cerevisiae*, 894 were common but 57 were strain-specific (Dowell *et al.* 2010). Furthermore, the detected LOF mutations constitute only a small fraction of all nonsynonymous changes. The remaining ones can code for a variety of defects, including complete or partial LOF, and also for adaptive or compensatory effects. In sum, it is straightforward to use whole-genome sequences to infer phylogenetic trees but much less so to identify phenotypically meaningful genetic polymorphisms.

We note that neither domesticated nor wild populations showed signs of significant outbreeding depression. Outbreeding depression has been found in a study involving crosses between yeast strains that formerly adapted to stressful conditions in the laboratory (Dettman *et al.* 2007). In our experiment, only in one test environment was progeny less fit than the parents. The agent applied there, benomyl, is not normally met in human-made habitats and is met even less in nature. It is unreasonable to expect that both parents adapted to it but in discordant ways. A recent review has confirmed that the depression is frequently caused by incompatibilities in the architecture of chromosomes (Frankham *et al.* 2011). Perhaps it is not incidental that in our experiment it was seen only when benomyl was present. This agent causes microtubule depolymerization and thus distracts mitosis, which could inflate the negative effect of any chromosomal discrepancies. Adaptations to different environments, if present and relevant to our test environments, were perhaps not conflicting. They also could have been lost when haploid strains were derived from the original diploid isolates (see *Materials and Methods*), especially if their effects depended on interactions within and between loci.

## Supplementary Material

Supporting Information
