# Supplementary material for: Heterosis Is Prevalent Among Domesticated but not Wild Strains of Saccharomyces cerevisiae
Source: G3 (Bethesda). 2013 Dec 16;4(2):315–23. doi: 10.1534/g3.113.009381 (PMC3931565; doi:10.1534/g3.113.009381)
Supplement: Supporting Information [file supp_4_2_315__index.html]

Heterosis Is Prevalent Among Domesticated but not Wild Strains of Saccharomyces cerevisiae — Supporting Information 

# Heterosis Is Prevalent Among Domesticated but not Wild Strains of *Saccharomyces cerevisiae*

## Supporting Information for Plech, de Visser, and Korona, 2014

**Files in this Data Supplement:**

- Supporting Information - Figures S1-S4 and Tables S1-S2 (PDF, 862 KB)
- Figure S1 - Correlation between the "intensity" scores of Colonyzer 2.0 and the cell counts (see Methods). (PDF, 241 KB)
- Figure S2 - All strains; correlation between the genetic distance and hybrid vigor measured as maximum growth rate (MGR), mean parent heterosis (MPH), and best parent heterosis (BPH). (PDF, 340 KB)
- Figure S3 - Domestic strains; correlation between the genetic distance and hybrid vigor measured as maximum growth rate (MGR), mean parent heterosis (MPH), and best parent heterosis (BPH). (PDF, 340 KB)
- Figure S4 - Wild strains; correlation between the genetic distance and hybrid vigor measured as maximum growth rate (MGR), mean parent heterosis (MPH), and best parent heterosis (BPH). (PDF, 355 KB)
- Table S1 - Liquid cultures; maximum growth rate (MGRs) of all homozygous and heterozygous strains in different test environments (see 'readme' sheet for legend). (.xls, 1 MB)
- Table S2 - Agar-surface cultures: average growth rate of agar cultures of all homozygous and heterozygous strains in different test environments (see 'readme' sheet for legend). (.xls, 1 MB)
