# Supplementary material for: Heterosis Is Prevalent Among Domesticated but not Wild Strains of Saccharomyces cerevisiae
Source: G3 (Bethesda). 2013 Dec 16;4(2):315–23. doi: 10.1534/g3.113.009381 (PMC3931565; doi:10.1534/g3.113.009381)
Supplement: Supporting Information [file supp_g3.113.009381_009381SI.pdf]

G3· Genes | Genomes | Genetics

Heterosis is prevalent among domesticated but not wild strains of *Saccharomyces cerevisiae*

Marcin Plech [*§], J. Arjan G.M. de Visser[§] and Ryszard Korona[*]

[*] Institute of Environmental Sciences, Jagiellonian University, 30-387 Krakow, Poland
[§] Laboratory of Genetics, Wageningen University, Wageningen, the Netherlands; phone

Corresponding Author:
Ryszard Korona
Institute of Environmental Sciences
Jagiellonian University
Gronostajowa 7
30-387 Krakow
Poland
phone (+)48126645136
e-mail: ryszard.korona@uj.edu.pl

DOI: 10.1534/g3.113.009381

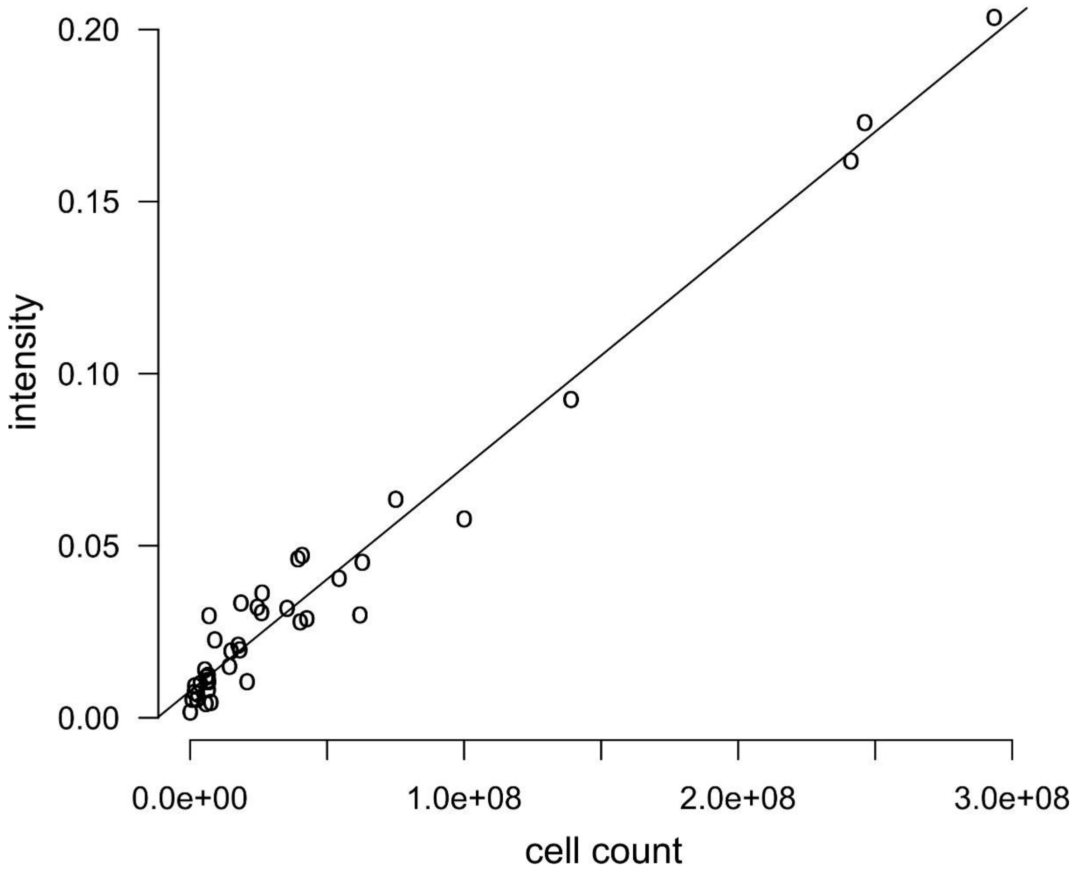

Figure S1 Correlation between the "intensity" scores of Colonyzer 2.0 and the cell counts (see Methods).

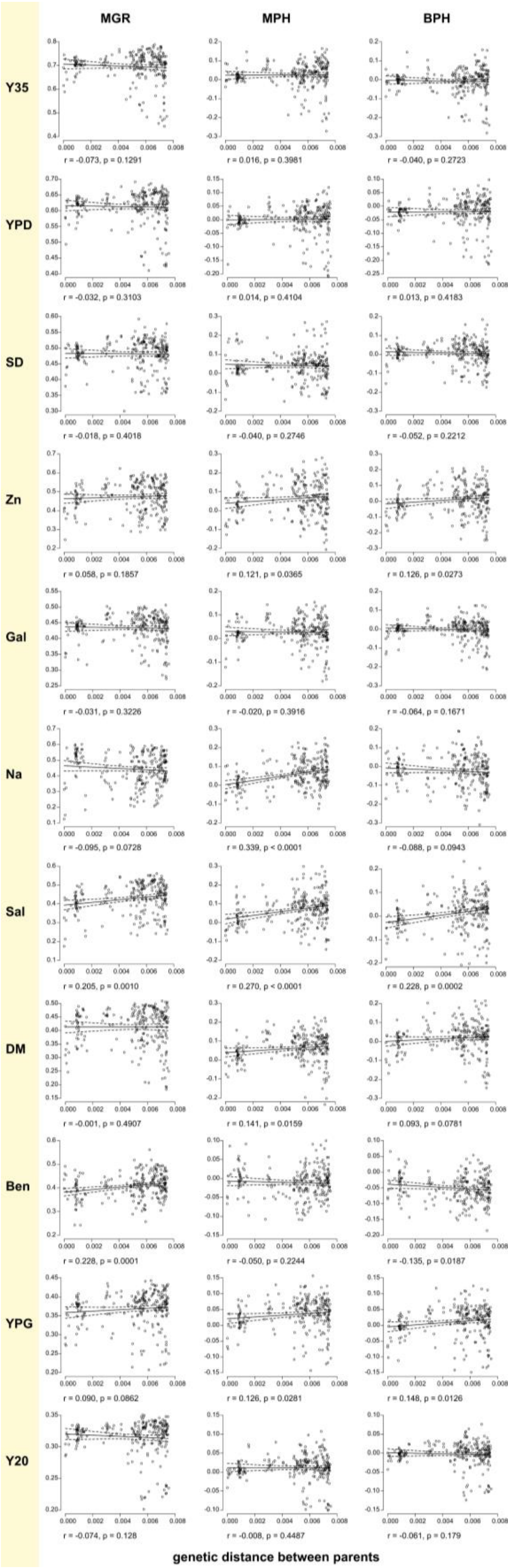

Figure S2 All strains; correlation between the genetic distance and hybrid vigor measured as maximum growth rate (MGR), mean parent heterosis (MPH), and best parent heterosis (BPH).

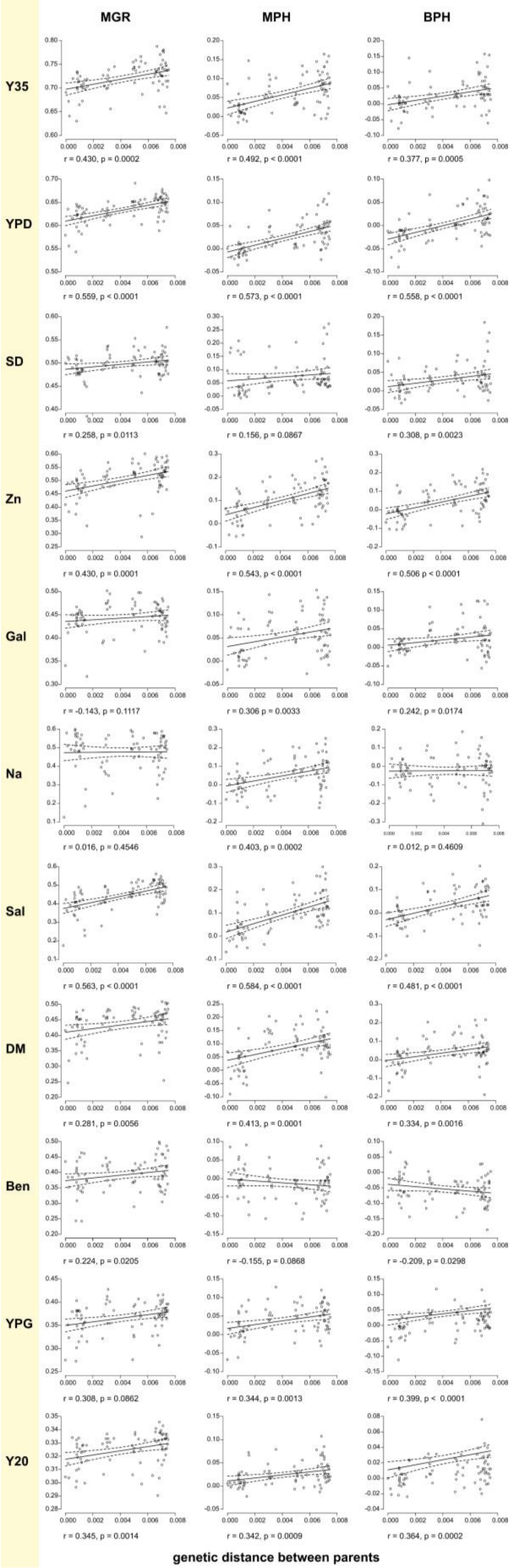

Figure S3 Domestic strains; correlation between the genetic distance and hybrid vigor measured as maximum growth rate (MGR), mean parent heterosis (MPH), and best parent heterosis (BPH).

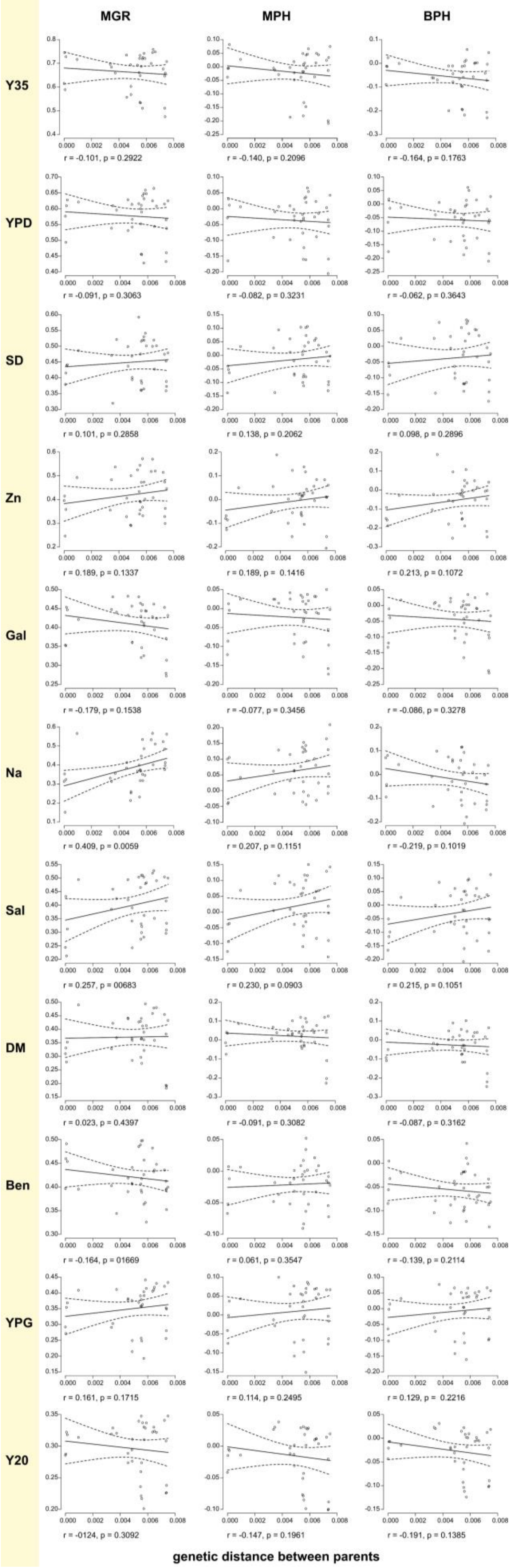
MGR
MPH
BPH
Y35
r = -0.101, p = 0.2922
r = -0.140, p = 0.2096
r = -0.164, p = 0.1763
YPD
r = -0.091, p = 0.3063
r = -0.082, p = 0.3231
r = -0.062, p = 0.3643
SD
r = 0.101, p = 0.2858
r = 0.138, p = 0.2062
r = 0.098, p = 0.2896
Zn
r = 0.189, p = 0.1337
r = 0.189, p = 0.1416
r = 0.213, p = 0.1072
Gal
r = -0.179, p = 0.1538
r = -0.077, p = 0.3456
r = -0.086, p = 0.3278
Na
r = 0.409, p = 0.0059
r = 0.207, p = 0.1151
r = -0.219, p = 0.1019
Sal
r = 0.257, p = 00683
r = 0.230, p = 0.0903
r = 0.215, p = 0.1051
DM
r = 0.023, p = 0.4397
r = -0.091, p = 0.3082
r = -0.087, p = 0.3162
Ben
r = -0.164, p = 01669
r = 0.061, p = 0.3547
r = -0.139, p = 0.2114
YPG
r = 0.161, p = 0.1715
r = 0.114, p = 0.2495
r = 0.129, p = 0.2216
Y20
r = -0124, p = 0.3092
r = -0.147, p = 0.1961
r = -0.191, p = 0.1385
genetic distance between parents

Figure S4 Wild strains; correlation between the genetic distance and hybrid vigor measured as maximum growth rate (MGR), mean parent heterosis (MPH), and best parent heterosis (BPH).

Tables S1-S2
Available for download at http://www.g3journal.org/lookup/suppl/doi:10.1534/g3.113.009381/-/DC1

Table S1 Liquid cultures; maximum growth rate (MGRs) of all homozygous and heterozygous strains in different test environments (see 'readme' sheet for legend).

Table S2 Agar–surface cultures: average growth rate of agar cultures of all homozygous and heterozygous strains in different test environments (see 'readme' sheet for legend).
