# Supplementary material for: Heterosis Is Prevalent Among Domesticated but not Wild Strains of Saccharomyces cerevisiae
Source: G3 (Bethesda). 2013 Dec 16;4(2):315–23. doi: 10.1534/g3.113.009381 (PMC3931565; doi:10.1534/g3.113.009381)
Supplement: Supporting Information [file supp_g3.113.009381_FigureS3.pdf]

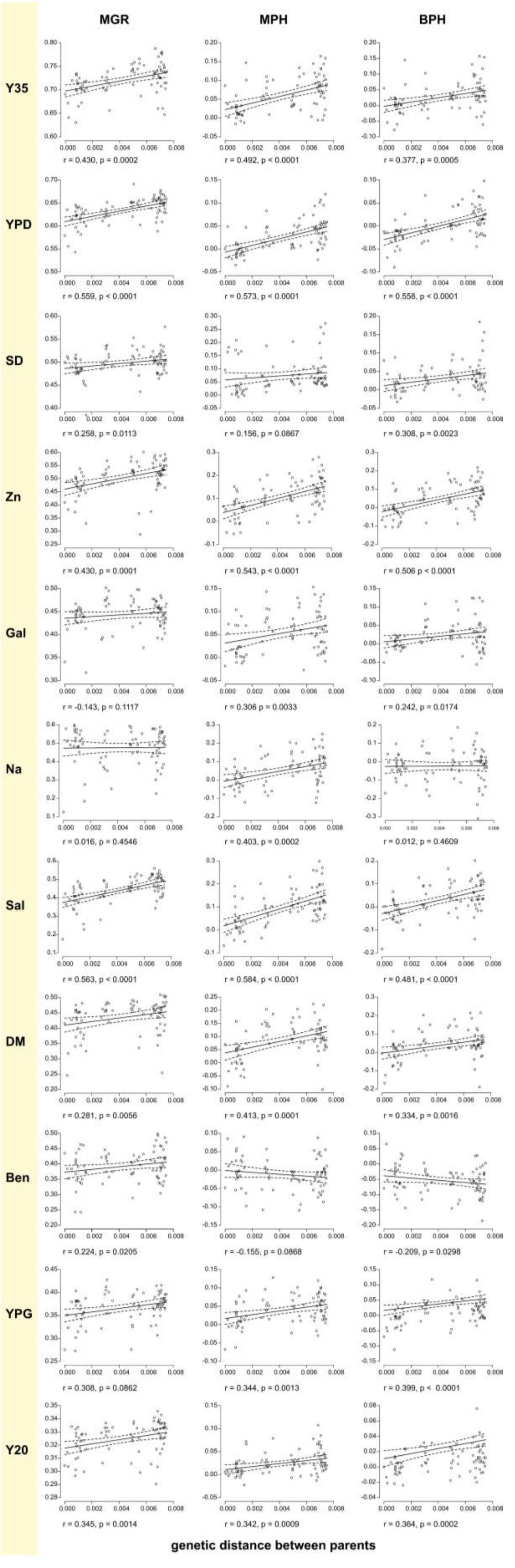

Figure S3 Domestic strains; correlation between the genetic distance and hybrid vigor measured as maximum growth rate (MGR), mean parent heterosis (MPH), and best parent heterosis (BPH).
