# Supplementary material for: Heterosis Is Prevalent Among Domesticated but not Wild Strains of Saccharomyces cerevisiae
Source: G3 (Bethesda). 2013 Dec 16;4(2):315–23. doi: 10.1534/g3.113.009381 (PMC3931565; doi:10.1534/g3.113.009381)
Supplement: Supporting Information [file supp_g3.113.009381_FigureS4.pdf]

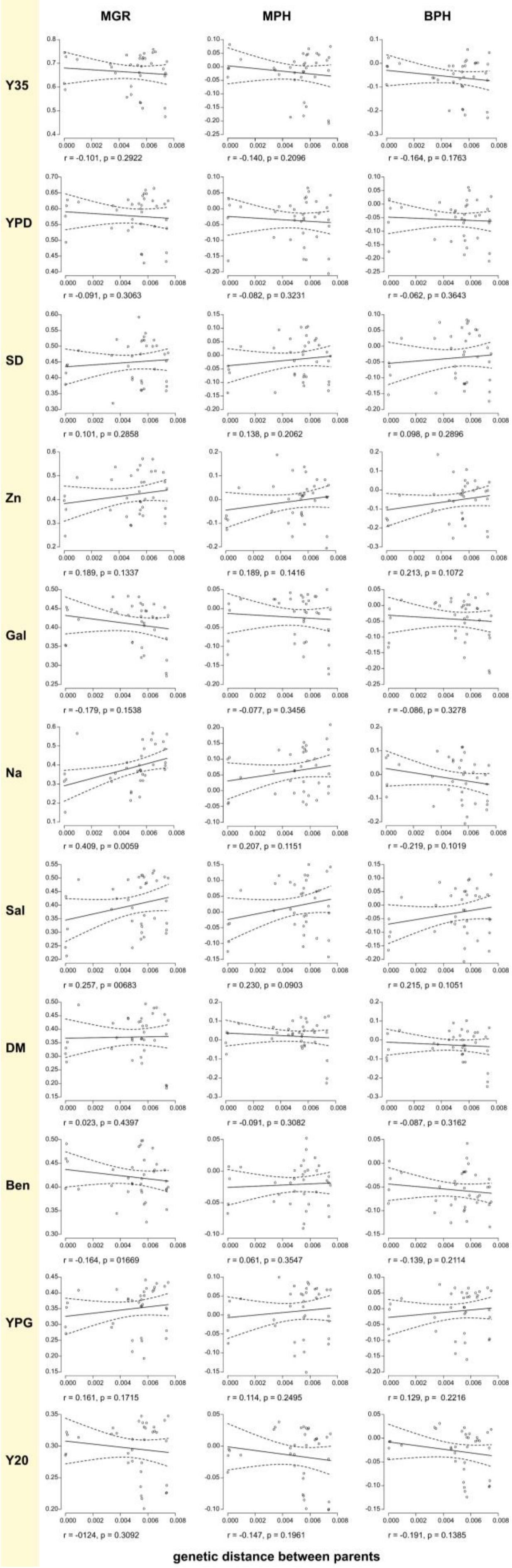
MGR
MPH
BPH
Y35
r = -0.101, p = 0.2922
r = -0.140, p = 0.2096
r = -0.164, p = 0.1763
YPD
r = -0.091, p = 0.3063
r = -0.082, p = 0.3231
r = -0.062, p = 0.3643
SD
r = 0.101, p = 0.2858
r = 0.138, p = 0.2062
r = 0.098, p = 0.2896
Zn
r = 0.189, p = 0.1337
r = 0.189, p = 0.1416
r = 0.213, p = 0.1072
Gal
r = -0.179, p = 0.1538
r = -0.077, p = 0.3456
r = -0.086, p = 0.3278
Na
r = 0.409, p = 0.0059
r = 0.207, p = 0.1151
r = -0.219, p = 0.1019
Sal
r = 0.257, p = 00683
r = 0.230, p = 0.0903
r = 0.215, p = 0.1051
DM
r = 0.023, p = 0.4397
r = -0.091, p = 0.3082
r = -0.087, p = 0.3162
Ben
r = -0.164, p = 01669
r = 0.061, p = 0.3547
r = -0.139, p = 0.2114
YPG
r = 0.161, p = 0.1715
r = 0.114, p = 0.2495
r = 0.129, p = 0.2216
Y20
r = -0124, p = 0.3092
r = -0.147, p = 0.1961
r = -0.191, p = 0.1385
genetic distance between parents

**Figure S4** Wild strains; correlation between the genetic distance and hybrid vigor measured as maximum growth rate (MGR), mean parent heterosis (MPH), and best parent heterosis (BPH).
